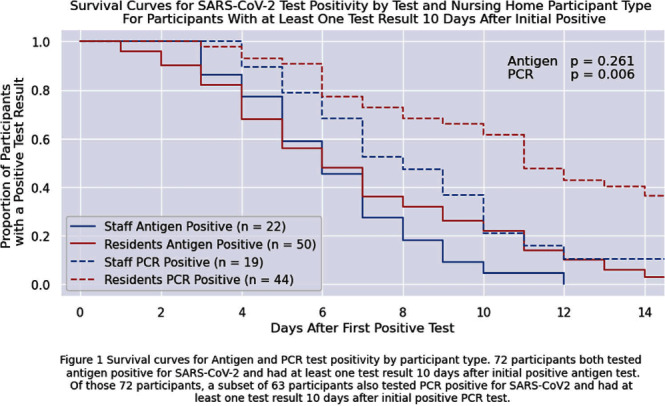# Viral Kinetics of SARS-CoV-2 in Nursing Home Residents and Staff

**DOI:** 10.1017/ash.2024.202

**Published:** 2024-09-16

**Authors:** Majerle Reeves, Scott Fridkin, Rachel Slayton, Yasin Abul, Christopher Crnich, Jazmin Duque, Jon Furuno, Stefan Gravenstein, Steven Handler, Jennifer Harcourt, Jessica Healy, Marc Lipsitch, Joseph Lutgring, Jennifer Meddings, Jennifer Meece, Lona Mody, David Nace, Prabasaj Paul, Paulina A. Rebolledo, Tiffany Harris, Morgan Katz, Sujan Reddy, David Canaday

**Affiliations:** Centers for Disease Control and Prevention; Emory Healthcare and Emory University; Centers for Disease Control and Prevention, Division of Healthcare Quality Promotion; University of Wisconsin; Oregon State University College of Pharmacy; Brown University and Providence Veterans Administration Medical Center; University of Michigan; VA Ann Arbor Healthcare System; University of Pittsburgh; Emory University School of Medicine; Johns Hopkins University

## Abstract

**Background:** Nursing home (NH) residents are at high risk of COVID-19 from exposure to infected staff and other residents. Understanding SARS-CoV-2 viral RNA kinetics in residents and staff can guide testing, isolation, and return to work recommendations. We sought to determine the duration of antigen test and polymerase chain reaction (PCR) positivity in a cohort of NH residents and staff. **Methods:** We prospectively collected data on SARS-CoV-2 viral kinetics from April 2023 through November 2023. Staff and residents could enroll prospectively or upon a positive test (identified through routine clinical testing, screening, or outbreak response testing). Participating facilities performed routine clinical testing; asymptomatic testing of contacts was performed within 48 hours if an outbreak or known exposure occurred and upon (re-) admission. Enrolled participants who tested positive for SARS-CoV-2 were re-tested daily for 14 days with both nasal antigen and nasal PCR tests. All PCR tests were run by a central lab with the same assay. We conducted a Kaplan-Meier survival analysis on time to first negative test restricted to participants who initially tested positive (day zero) and had at least one test ≥10 days after initially testing positive with the same test type; a participant could contribute to both antigen and PCR survival curves. We compared survival curves for staff and residents using the log-rank test. **Results:** Twenty-four nursing homes in eight states participated; 587 participants (275 residents, 312 staff) enrolled in the evaluation, participants were only tested through routine clinical or outbreak response testing. Seventy-two participants tested positive for antigen; of these, 63 tested PCR-positive. Residents were antigen- and PCR-positive longer than staff (Figure 1), but this finding is only statistically significant (p=0.006) for duration of PCR positivity. Five days after the first positive test, 56% of 50 residents and 59% of 22 staff remained antigen-positive; 91% of 44 residents and 79% of 19 staff were PCR-positive. Ten days after the first positive test, 22% of 50 residents and 5% of 22 staff remained antigen-positive; 61% of 44 residents and 21% of 19 staff remained PCR-positive. **Conclusions:** Most NH residents and staff with SARS-CoV-2 remained antigen- or PCR-positive 5 days after the initial positive test; however, differences between staff and resident test positivity were noted at 10 days. These data can inform recommendations for testing, duration of NH resident isolation, and return to work guidance for staff. Additional viral culture data may strengthen these conclusions.

**Disclosure:** Stefan Gravenstein: Received consulting and speaker fees from most vaccine manufacturers (Sanofi, Seqirus, Moderna, Merck, Janssen, Pfizer, Novavax, GSK, and have or expect to receive grant funding from several (Sanofi, Seqirus, Moderna, Pfizer, GSK). Lona Mody: NIH, VA, CDC, Kahn Foundation; Honoraria: UpToDate; Contracted Research: Nano-Vibronix

**Figure:**